# Macrophage and T-Cell Infiltration and Topographic Immune Cell Distribution in Non-Melanoma Skin Cancer of the Head and Neck

**DOI:** 10.3389/fonc.2022.809687

**Published:** 2022-04-07

**Authors:** Gesche Frohwitter, Marie Kerta, Christoph Vogl, Carol Immanuel Geppert, Jan-Erik Werry, Jutta Ries, Marco Kesting, Manuel Weber

**Affiliations:** ^1^ Department for Oral and Maxillofacial Surgery, University Hospital Erlangen, Friedrich-Alexander-University Erlangen-Nürnberg, Bavaria, Germany; ^2^ Institute of Pathology, University Hospital Erlangen, Friedrich-Alexander-University Erlangen-Nürnberg, Bavaria, Germany

**Keywords:** non-melanoma skin cancer, macrophages, T cells, cancer immunology, immunotherapy, checkpoint

## Abstract

Non-melanoma skin cancer (NMSC) is a heterogeneous tumor entity that is vastly determined by age and UV-light exposure leading to a great mutational burden in cancer cells. However, the success of immune checkpoint blockade in advanced NMSC and the incidence and disease control rates of NMSC in organ transplant recipients compared to immunologically uncompromised patients point toward the emerging importance of the immunologic activity of NMSC. To gain first insight into the role of T-cell and macrophage infiltration in NMSC of the head and neck and capture their different immunogenic profiles, which appear to be highly relevant for the response to immunotherapy, we conducted a whole slide analysis of 107 basal cell carcinoma (BCC) samples and 117 cutaneous squamous cell carcinoma (cSCC) samples. The CD8^+^ and CD68^+^ immune cell expression in both cancer types was evaluated by immunohistochemistry and a topographic distribution profile, and the proportion of both cell populations within the two tumor entities was assessed. The results show highly significant differences in terms of CD8^+^ T-cell and CD68^+^ macrophage infiltration in BCC and cSCC and indicate cSCC as a highly immunogenic tumor. Yet, BCC presents less immune cell infiltration; the relation between the immune cells compared to cSCC does not show any significant difference. These findings help explain disparities in local aggressiveness, distant metastasis, and eligibility for immune checkpoint blockade in both tumor entities and encourage further research.

## Introduction

Non-melanoma skin cancer (NMSC) includes basal cell carcinoma (BCC) and cutaneous squamous cell carcinoma (cSCC). Whereas BCC is the most common cancer in humans, cSCC represents the second most common type of NMSC, with a constantly rising incidence worldwide and a predisposition at the sun-exposed skin of the head and neck (80% of all NMSCs) (1, 2). Emerging research emphasizes the significant influence of the immune system on NMSC development and suggests a strong association to the therapeutic response and prognosis beyond the TNM classification (3). These trends go in line with the fact that immunosuppression increases cSCC incidence rate by 65 to 250 times, with cSCC being the most common cancer in solid organ transplant recipients (4). Furthermore, immune response modifiers show beneficial effects in precancerous skin lesions and NMSC and compete with surgical treatment in cases of field cancerization (5). Even though these findings suggest a strong association between the immune system and cSCC, little knowledge on immunological aspects of tumor development and prognosis exists in this field. The admission of immune checkpoint inhibitors [e.g., anti-programmed cell death-1/programmed cell death ligand 1 (PD-1/PD-L1) antibodies] in primary and advanced cancer had a striking effect on progressive-free survival, overall survival, and objective response rates on various types of cancer (6–9). In advanced cases of cSCC with missing surgical and radio-oncological curative treatment options, immunotherapy with the PD-1 inhibitor cemiplimab is standard of care now (10). The response rate of up to 50% in locally advanced cases and 47% in metastatic cases provides success rates beyond any other palliative treatment option in cSCC. Moreover, even though BCC progression is primarily mediated by the Sonic Hedgehog pathway (HP) and its tumor mutational burden appears to be significantly lower than that of cSCC, BCC has been shown to respond to PD-L1 inhibition as well (11). Therefore, the PD-1 inhibitor cemiplimab was recently approved for the treatment of advanced BCC (12).

Even though the overall prognosis for NMSC is gratifying, its clinical behavior in terms of local aggressiveness, metastatic spread, and recurrence differs significantly at a disadvantage to advanced cSCC cases. Although cSCC tumor mutational burden is among the highest in solid tumors, the immunogenicity appears to vary between BCC and cSCC (13, 14). By evading immune surveillance through cell impairment, T-cell function and macrophage polarization were shown to be players in tumor development and progression (15–17). Furthermore, T cells are part of the adaptive immune system and represent one of the best-characterized immune cell types in cancer and are the key effector cells for antitumor immune reactions (15, 16). As macrophages are highly plastic and part of the innate immune system, their role as antigen-presenting cells is critical for the initiation of specific antitumor T-cell responses (18, 19).

Recent findings suggest an impaired immunosurveillance in BCC that show a reduced major histocompatibility complex I (MHC-I) expression with a low CD4^+^ and CD8^+^ T-cell infiltration and an increase in regulatory T-cell (T_reg_) infiltration with an uprise in interleukins and cytokines that promote immunosuppression (13, 14). In organ transplant recipients, a high expression of a CD57-expressing CD8^+^ T-cell subtype was a strong predictor for the development and recurrence of cSCC (20). Additionally, the differentiation of T-lymphocyte profiles in cSCC has shown that moderately and poorly differentiated cSCC had a higher PD-1/PDL-1 expression that correlated with an increased number of CD4^+^, CD8^+^, CD4^+^ FOXp3^+^ regulatory T cells and enhanced tumor invasiveness (21). Besides the clinical significance of T lymphocytes, macrophages are closely related to inflammatory disease and tumor outcome (17, 22–24). In renal cell carcinoma, the expression of macrophage markers (CD163, CD203, CD206) in mass cytometry with antibody panels was associated with an increased expression of PD-1 on CD8^+^ T cells, which suggest a direct interplay of myeloid and lymphoid cell lines in tumor progression and may serve as a common therapeutic target (25). Furthermore, in mucosal SCC, macrophage infiltration was associated with lymph node metastases and survival (17, 23).

To gain first insight into the role of T-cell and macrophage infiltration in NMSC of the head and neck and capture their different immunogenic profiles, which appear to be highly relevant for the therapeutic response of immunotherapy, we conducted a whole slide analysis of NMCS samples (BCC and cSCC). The CD8^+^ and CD68^+^ immune cell expression in both cancer types was evaluated, and a topographic distribution profile as well as the proportion of both cell populations within the two tumor entities was assessed.

## Materials and Methods

### Patients and Tissue Harvesting

The retrospective analysis was composed of tissue specimens from 107 BCC and 117 cases of cSCC that were treated at the Department for Oral and Maxillofacial Surgery at the University Hospital Erlangen during 2010 and 2020. The study was approved by the local ethics committee of the Friedrich-Alexander University Erlangen-Nuremberg (54_17Bc) and performed in accordance with the Declaration of Helsinki.

The resected specimens of both BCC and cSCC were determined by the histopathological reports and the visual tumor inspection under light microscope (Axio Imager 2, Carl Zeiss, Germany). From each tumor specimen, 2-µm sections were prepared using a rotary microtome (Leica, Nussloch, Germany). The prepared tumor sections were subsequently processed for immunohistochemistry.

### Immunohistochemical Staining

Sections were deparaffinized with xylene and then rehydrated through a descending alcohol series. Epitope unmasking was performed by heat-induced epitope recovery. For this purpose, samples were heated in citrate (pH 6.0; CD68) buffer for 30 min each or EDTA buffer (pH 9.0; CD8) for 20 min each and allowed to rest at room temperature. For immunohistochemical staining, the polymer detection method by immunostaining (Autostainer Plus, Dako cytomation, Aligent, Santa Clara, USA) was applied. The immunohistochemical stainings were performed as previously described (17, 24). The following primary antibodies were used: anti-CD8 (M7103; host mouse; DAKO, Glostrup, Denmark; dilution 1:100) and anti-CD68 (M0814; host mouse; DAKO, Glostrup, Denmark; dilution 1:3,000).

For visualization, the Histofine Simple Stain MAX PO staining kit (DAB kit, medac, Wedel, Germany) was used according to the recommendation of the manufacturer. Counterstaining of the samples was done using hematoxylin (DAKO, Glostrup, Denmark). A section of a human tonsil was included as a positive control to evaluate the staining results. Tissue samples without application of the primary antibody were used as negative controls.

### Digitalization and Statistical Analysis

**Figure 1:**
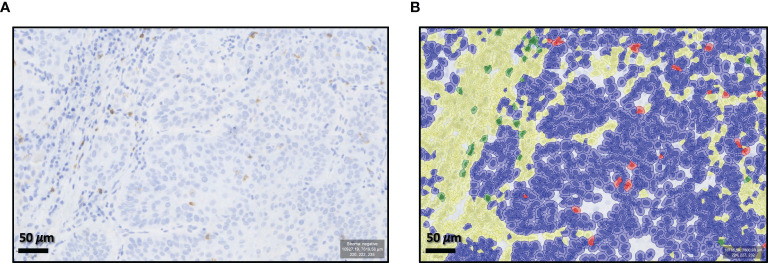
Qu-Path-0.2.0 analysis software training. Example of “Positive Cell Detection” by the freeworks analysis software Qu-Path-0.2.0. **(A)** Shows the unmarked slide (×70 magnification). **(B)** Shows the automatic classifier division for cd8^+^- or cd68^+^-positive cells and CD8^+^- or CD68^+^-negative cells in the epithelial tumor compartment and the tumor stroma.

Digitization of the immunohistochemically stained sections was performed in cooperation with the Institute of Pathology at the University Hospital Erlangen (Scanner Pannoramic 1000/Scanner Pannoramic 250 Flash III, 3D Histech, Budapest, Hungary). The freeware analysis software QuPath-0.2.0 was used to analyze the sections (26). “Positive Cell Detection” was performed after manual labeling of the complete tumor area in each specimen. For this purpose, the following settings were changed compared to the default settings: Detection image: Optical density sum; Requested pixel size: 0.25 µm; Background radius: 8 µm, in case of insufficient cell detection 0 µm; Score compartment: Nucleus: DAB OD mean. Subsequently, a classifier was trained for each individual specimen. After manual selection of characteristic cells, the classifier automatically divided the remaining labeled tumor into the following cells: CD8- or CD68-positive cells and CD8- or CD68-negative cells. This was performed in the epithelial tumor compartment and the tumor stroma. Exclusion of artifacts was achieved by deleting inappropriate cells and cell regions and/or training the classifier. Thereafter, automatic cell counting (positive and negative cells) of the complete available tumor area was done (**Figure 1**).

For the invasion front, hotspot analysis of up to two prominent regions was performed: For this purpose, a “Positive Cell Detection” was performed with the following settings: Detection image: Optical density sum; Requested pixel size: 0.25 µm; Background radius: 8 µm; Score compartment: Nucleus: DAB OD max. Otherwise, the default settings were used. Differentiation of cells in the invasion front was limited to positive and negative cells in the respective CD8 or CD68 stained section.

The immunohistochemical stainings were analyzed by determining the cell count as the number of positively stained cells per mm^2^ of the specimen. The labeling index was calculated by dividing the number of positive cells by the number of all cells (positive + negative) counted in the tumor epithelium, the tumor stroma, and the tumor epithelium and stroma together. The results are displayed as the median and standard deviation (SD). The box plot diagrams represent the median, interquartile range, and minimum (min) and maximum (max). Two-sided adjusted p-values ≤0.05 were considered significant. The analyses were performed by the Mann–Whitney–U-Test with IBM SPSS Statistics Version 24 (Released 2016, IBM SPSS Statistics for Windows, IBM Corp., Armonk, NY).

## Results

### Clinicopathological Results

The gender distribution as well as the age distribution was equal between the two groups (68 men, 39 women, mean age 79.9 years in BCC and 80 men, 37 women, mean age 79.8 years in cSCC). Tumor staging was performed regarding the tumor (T), nodes (N), and metastases (M) (TNM) classification of the year 2017. The tumor sizes in BCC and cSCC patients were alike, with mainly T1 carcinomas (48.6% BCC, 47.0% cSCC). As expected, no nodal invasion (N-status) could be observed in BCC, but 9.4% of all cSCC showed local lymph node metastasis. The tumor grading in cSCC showed mostly G2 tumors (40.2%) followed by G3 tumors (32.5%) and G1 tumors (21.4%); only one (0.9%) tumor was classified as G4. The grading system was not applied to BCC. One BCC patient received an imiquimod therapy prior to surgical resection that was designated as neoadjuvant treatment. One cSCC patient received interstitial brachytherapy prior to tumor resection. However, adjuvant treatment was administered to 20 patients (18.7%) with a BCC and 9 patients (7.7%) with a cSCC. In this study, 22 patients were treated with adjuvant radiotherapy and 7 with adjuvant radiochemotherapy. Furthermore, the majority in both groups did not suffer from disease recurrence (BCC 88.8%, cSCC 79.5%), but more than half of all patients developed another malignant tumor (BCC 55.1%, cSCC 59.8%). Immunosuppression was more frequent in cSCC (9.4%) compared to BCC (0.9%) but played a minor role within the patient collective. Further information on the demographic characteristics is given in **Table 1**.

**Table 1:** Shows the demographic parameters of the patient collective.

TABLE 1	Description of the patient collective; total number of cases: 224				
		BCC		cSCC	
		*n*	*% of cases*	*n*	*% of cases*
**Number of cases**		107	100%	117	100%
**Gender**	*Men*	68	63.6%	80	68.4%
	*Women*	39	36.4%	37	31.6%
**Age**	*Mean*	72.9		79.8	
	*Range*	20.17–95.85		45.37–100.47	
**TNM–tumor size**	*T1*	52	48.6%	55	47.0%
	*T2*	4	3.7%	23	19.7%
	*T3*	2	1.9%	22	18.8%
	*T4*	2	1.9%	1	0.9%
	*Tx*	47	43.9%	16	13.7%
**TNM–nodal invasion**	*N0*	17	15.9%	45	38.5%
	*N1*	0	0%	4	3.4%
	*N2*	0	0%	3	2.6%
	*N3*	0	0%	4	3.4%
	*Nx*	90	81.4%	61	52.1%
**TNM–distant metastasis**	*M0*	17	15.9%	69	59.0%
	*M1*	0	0%	2	1.7%
	*Mx*	90	84.1%	46	39.3%
**TNM–grading**	*G1*	0	0%	25	21.4%
	*G2*	0	0%	47	40.2%
	*G3*	0	0%	38	32.5%
	*G4*	0	0%	1	0.9%
	*Gx*	107	100%	6	5.1%
**Neoadjuvant therapy**	*Yes*	1	0.9%	1	0.9%
	*No*	104	97.2%	115	98.3%
	*Unknown*	2	1.9%	1	0.9%
**Adjuvant therapy**	*Yes*	20	18.7%	9	7.7%
	*No*	73	68.2%	56	47.9%
	*Unknown*	14	13.1%	52	44.4%
**Recurrence**	*Yes*	11	10.3%	12	10.3%
	*No*	95	88.8%	93	79.5%
	*Unknown*	1	0.9%	12	10.2%
**Immunosuppression**	*Yes*	1	0.9%	11	9.4%
	*No*	101	94.4%	102	87.2%
	*Unknown*	5	4.7%	4	3.4%
**Other tumor diseases**	*Yes*	59	55.1%	70	59.8%
	*No*	43	40.2%	47	40.2%
	*Unknown*	5	4.7%	0	0.00%

BCC, basal cell carcinoma; cSCC, cutaneous squamous cell carcinoma.

Gender, age at diagnosis, the TNM classification, grading, (neo-)adjuvant treatments, recurrence, other tumor disease, and immune status are displayed.

### Distribution of Macrophage and T-Cell Infiltration in Basal Cell Carcinoma and Cutaneous Squamous Cell Carcinoma

**Figure 2:**
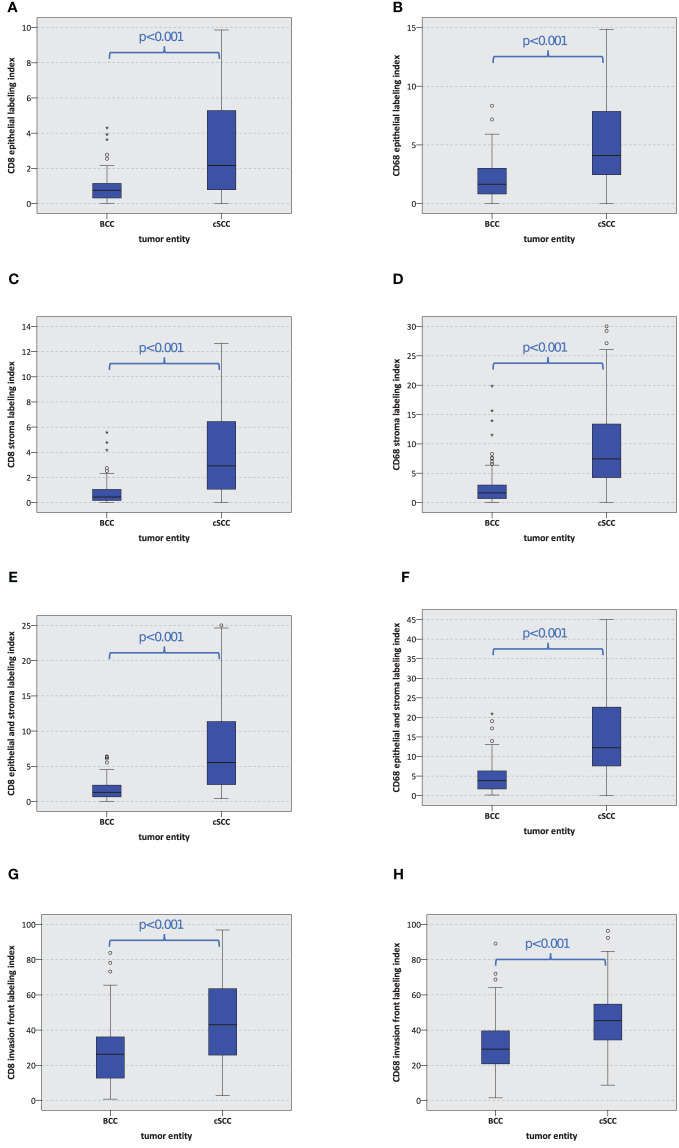
Macrophage and T-cell expression. The box plots show the labeling index (percentage of expressing cells) for CD8^+^ and CD68^+^ cells in basal cell carcinoma (BCC) and cutaneous squamous cell carcinoma (cSCC) **(A–H)**. The p-values are generated by the Mann–Whitney U test.

The CD8 and CD68 labeling indexes were significantly lower in BCC compared to cSCC (p < 0.001) (**Table 2** and **Figures 2A–H**). These results hold true for all tumor regions, namely, the tumor epithelium, the tumor stroma, the entire tumor (epithelium and stroma), and the tumor invasion front. In the epithelial tumor compartment, the median CD8 labeling index in cSCC was 2.17% compared to 0.75% in BCC (**Table 2**). Epithelial CD68 labeling index was 4.09% in cSCC compared to 1.65 in BCC (**Table 2**).

**Table 2:** Shows the T-cell (CD8) and macrophage (CD68) labeling index (LI) and cell count (positive cells/mm^2^) in basal cell carcinoma (BCC) and cutaneous squamous cell carcinoma (cSCC) in the epithelium, stroma, and tumor invasion front.

TABLE 2	Macrophage cell labeling index and count (cells/mm^2^) in basal cell carcinoma (BCC) and cutaneous squamous cell carcinoma (cSCC)							
Group/Marker		CD8				CD68		
	n	Median	SD	p-value	n	Median	SD	p-value
***Epithelial labeling index (ELI)***								
BCC	107	0.75	0.78	***<0.001***	107	1.65	1.53	***<0.001***
cSCC	114	2.17	4.44		115	4.09	4.69	
***Stroma labeling index (SLI)***								
BCC	107	0.44	0.93	***<0.001***	107	1.65	3.15	***<0.001***
cSCC	114	2.92	5.86		115	7.44	8.28	
***Epithelial and stroma labeling index (ESLI)***								
BCC	107	1.30	1.31	***<0.001***	107	3.8500	3.81	***<0.001***
cSCC	114	5.55	9.08		115	12.25	10.55	
***Invasion front labeling index (IFLI)***								
BCC	107	26.26	17.67	***<0.001***	107	29.18	16.19	***<0.001***
cSCC	85	43.04	23.02		73	45.36	19.20	
***Epithelial cells/mm^2^***								
BCC	107	53.01	69.96	*0.001*	107	128.27	126.65	***<0.001***
cSCC	116	85.13	266.13		115	206.56	296.81	
***Stroma cells/mm^2^***								
BCC	107	35.08	60.22	***<0.001***	107	129.73	173.19	***<0.001***
cSCC	114	115.54	343.08		115	414.81	502.25	
***Epithelial and stroma cells/mm^2^***								
BCC	107	98.64	99.26	***<0.001***	107	282.17	237.37	***<0.001***
cSCC	114	234.82	538.61		115	683.57	688.46	
***Invasion front cells/mm^2^***								
BCC	107	1,652.80	1,579.16	***<0.001***	107	2,159.56	1,355.20	***<0.001***
cSCC	85	3,469.61	1,912.20		73	4,218.09	1,737.27	

Values represent the median, standard deviation (SD), and p-value (Mann–Whitney U test). n, number of cases. Significant p-values are printed in bold letters.

The tumor invasive front showed the highest infiltration of both immune cells. Median CD8 labeling index at the invasive front of cSCC was 43.04% compared to 26.26% in BCC. At the invasive front of cSCC, 45.36% of the cells showed CD68 expression compared to 29.18% in BCC. The details are given in **Table 2** and displayed in **Figures 2A–H**.

**Figure 3:**
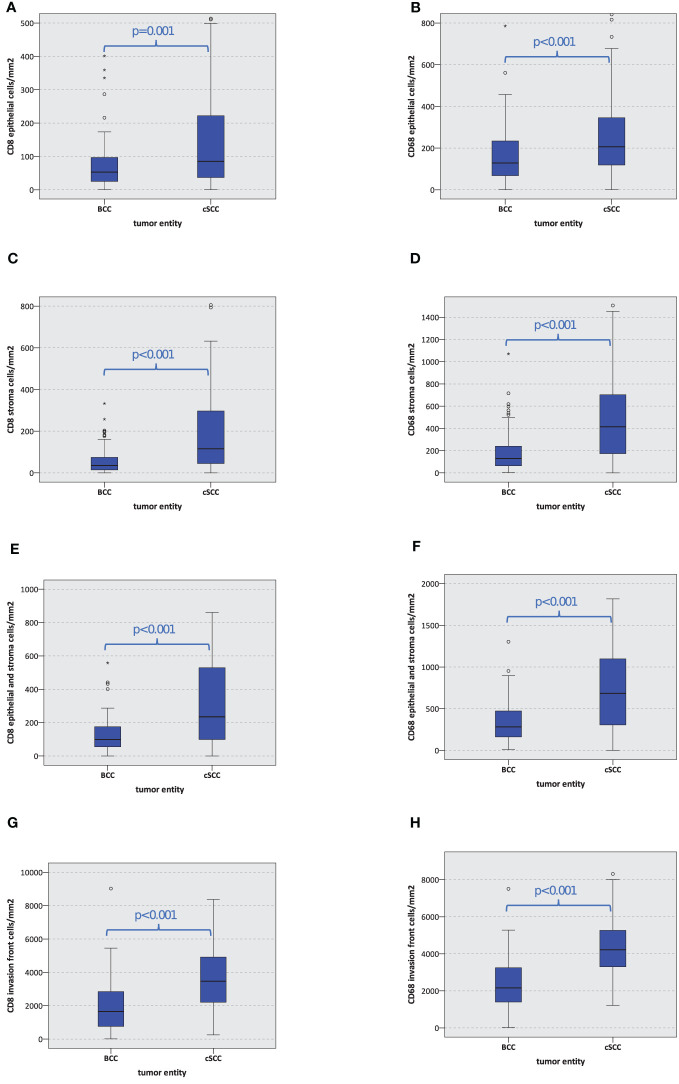
Macrophage and T-cell infiltration. The box plots show macrophage and T-cell infiltration (positive cells/mm^2^) for CD8 and CD68 in basal cell carcinoma (BCC) and cutaneous squamous cell carcinoma (cSCC) **(A–H)**. The p-values are generated by the Mann–Whitney U test.

Furthermore, the absolute number of CD8- and CD68-positive cells per mm^2^ in BCC was significantly lower compared to cSCC (p ≤ 0.001) in the tumor epithelium, the tumor stroma, the entire tumor (epithelium and stroma), and the tumor invasion front. The details are given in **Table 2** and displayed in **Figures 3A–H**.

**Figure 4:**
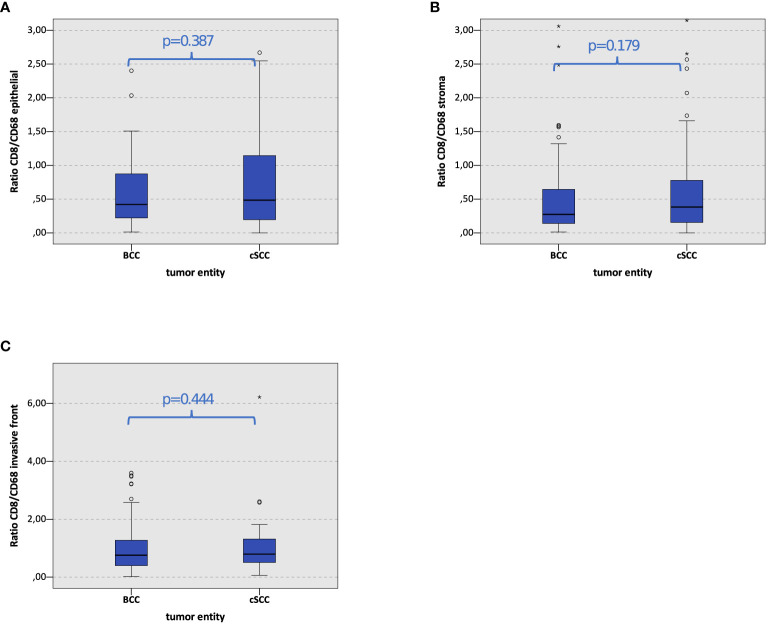
Expression patterns for CD8^+^ and CD68^+^ basal cell carcinoma (BCC) and cutaneous squamous cell carcinoma (cSCC). Typical expression patterns of CD8^+^ and CD68^+^ in BCC and cSCC. The fields of view show a panoramic image (×30 magnification) and a high-power image (×70 magnification). Examples of staining patterns for CD8^+^ and CD68^+^ for the tumor center and the invasion front of both tumor entities are given. The BCC micrograph slides are identical in all images and show a stronger staining pattern in the tumor invasion front compared to the tumor center for both CD8^+^ and CD68^+^. The cSCC micrographs show a similar expression pattern as the BCC slides. For cSCC, all micrographs are taken from one slide but from different regions.

Examples of characteristic staining patterns are given in **Figures 4**. The BCC micrograph slides are matching in all images and show a stronger staining pattern in the tumor invasion front compared to the tumor center for both CD8 and CD68. The cSCC micrographs show a similar expression pattern as the BCC slides, however, with overall higher cell density.

### Relation of T-Cell Expression Compared to Macrophage Expression in Basal Cell Carcinoma and Cutaneous Squamous Cell Carcinoma

**Figure 5:**
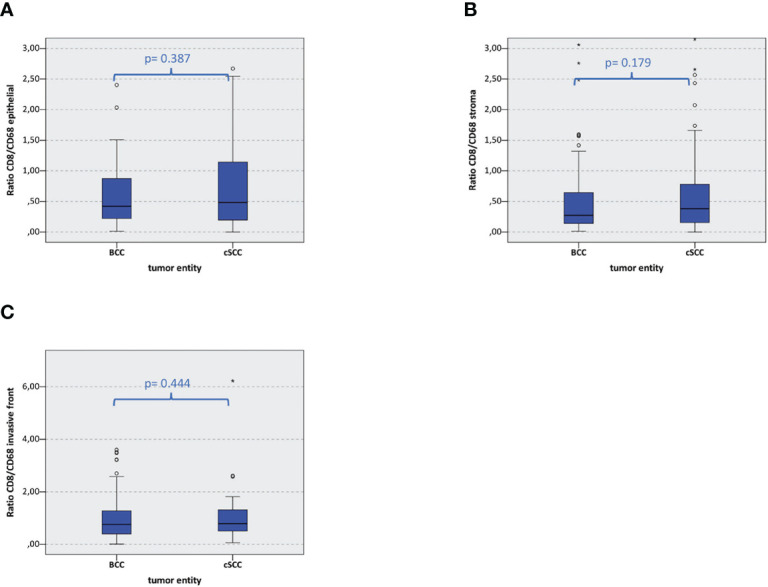
Macrophage and T-cell infiltration. The box plots show CD8/CD68 ratio in basal cell carcinoma (BCC) and cutaneous squamous cell carcinoma (cSCC) in the epithelial tumor compartment, the tumor stroma, and the tumor invasion front **(A–C)**. The p-values are generated by the Mann–Whitney U test.

The relation of the overall CD8^+^ stained cells to the amount of CD68^+^ stained cells was calculated in BCC and cSCC. No significant difference between the relation of the CD8^+^ T cell and CD68^+^ macrophage cell count could be found in BCC compared to cSCC (**Figure 5**). The relation of CD8^+^ T cells to CD68^+^ macrophages remains the same in both carcinomas.

### Distribution of Immune Cell Infiltration in Different Tumor Compartments

**Figure 6:**
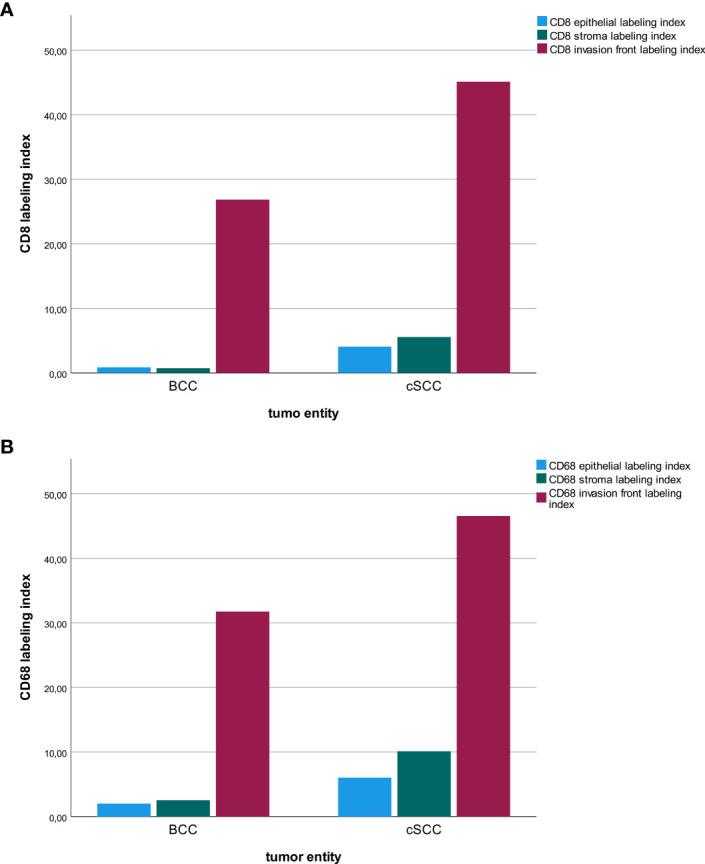
Macrophage and T-cell infiltration. The diagrams show a comparison of the labeling index (percentage of expressing cells) for CD8^+^ **(A)** and CD68^+^ **(B)** cells regarding the tumor location (tumor center epithelial, tumor center stroma, tumor invasion front) in basal cell carcinoma (BCC) and cutaneous squamous cell carcinoma (cSCC).

The tumor invasion front showed by far the highest immune cell infiltration compared to the epithelial tumor compartment and the stroma in the tumor center. This was observable in BCC and in cSCC (**Figure 6**). However, it needs to be considered that for the invasive front, a hot-spot analysis of regions with the highest immune cell infiltration was performed, while the epithelium and tumor stroma were counted on a whole-slide level.

## Discussion

The results of our research confirm a relevant immune cell infiltration in both BCC and cSCC. However, the higher CD8^+^ and CD68^+^ infiltration in all tumor regions, namely, the tumor epithelium, the tumor stroma, the entire tumor (epithelium and stroma), and the tumor invasion front was more distinct in cSCC compared to that in BCC. These findings outline that immunologic pressure might play a more significant role in cSCC compared to BCC. Our results are consistent with previous data also showing increased CD8 infiltration in cSCC compared to BCC (27). There are further data indicating increased CD68 infiltration in cSCC compared to BCC (28).

With a view to the tumor subregions, the invasion front showed the highest infiltration of both immune cell types, CD8^+^ T cells and CD68^+^ macrophages, compared to the stroma and the epithelium in both NMSCs. This finding is interesting, as the tumor front is presumed to contain a more aggressive cell phenotype, known as leader cells that promote tissue invasion and ease the way for cancer cell spread (29, 30). Here, the interface between the tumor and the host is most active and the immune cell infiltration at the tumor invasion front and the tumor microenvironment is a key player in therapy response to immune checkpoint blockade (31). Our results show that the highest CD8^+^ T-cell infiltration can be found at the tumor invasion front in both BCC and cSCC, which underlines their potential immunogenic activity. These results go in line with the prognostic relevance of the CD3^+^ and CD8^+^ T-cell infiltration in the tumor and the invasive tumor margin in colon cancer, where patients with a high immunoscore show the lowest 5-year disease recurrence (32). The measurements of CD3^+^ and CD8^+^ T cells revealed a high reproductivity between the groups of different scientists and were implied in the TNM classification as a highly relevant prognostic factor (32).

As cytotoxic CD8^+^ T cells can detect antigens by MHC-I activation that consequently leads to an elimination of neoplastic and infected cells, the response rates of PD-1 inhibitors in immunogenic T cell-enriched tumors as the cSCC come as no surprise. *Vice versa*, it is known that a dominant activation of the PD-1/PD-L1 axis in cancer leads to a downregulation in T-cell activity that increases tumor aggressiveness (33).

It is shown that BCC development is strongly associated with aberrations in the HP. This can be targeted using the HP inhibitor vismodegib, which is approved for patients who are not suitable for surgical or radio-oncological treatment. As an additional pharmacologic treatment option, cemiplimab has recently been approved by the European Medicines Agency (EMA) and US Food and Drug Administration (FDA) as a treatment option when vismodegib has resulted in an inappropriate treatment response (12, 34). Remarkably, the application of vismodegib may even lead to an upregulation of MHC-I on BCC tumor cells and an increased infiltration of CD4^+^, CD8^+^, and HLA-DR-class II-positive cells like macrophages within the tumor cell nests (35). Furthermore, an upregulation in CD8^+^ T cells in immunologically deprived BCC and cSCC could be observed by the topical application of the immunomodulator imiquimod that activates the tumor microenvironment in the presentation of MHC-I molecules (13, 36). These results and our findings confirm a less dominant role of immunosurveillance in BCC compared to cSCC but at the same time reveal their immunogenic potential. As a high CD8^+^ infiltration is associated with a favorable response to immune checkpoint blockade and an improved outcome, the positive effect of cemiplimab treatment in NMSC is conclusive (32, 37). CD8 infiltration seems also to increase in cSCC during malignant transformation. An increased CD8 infiltration in cSCC compared to normal skin and actinic ceratosis is shown (38). In addition, the systemic status of immune competence seems to be of importance. In cSCC tumors of organ transplant recipients, a significantly reduced CD8 infiltration was found (39). In the current patient collective, only one patient was immunosuppressed and it therefore represents a relatively homogeneous cohort in this regard.

When it comes to tumor macrophages, we could observe similar results for CD68^+^ cells compared to those of the CD8^+^ T-cell group with a significantly more dominant expression in cSCC compared to BCC. Macrophages are known to be highly plastic and are part of the tumor microenvironment in many cancers (22, 40, 41). Their ability to switch between an “antitumor” M1 polarized state in which they promote antitumor immunity and eliminate pathogens and a “tumor-promoting” M2 polarization supporting angiogenesis and tumor progression makes them a highly attractive therapeutic target in immunologic research (17, 22–24, 42). As tumor-promoting M2 polarized macrophages are associated with an increased expression of PD-L1 in solid cancer, they may serve as a prognostic marker for immune checkpoint therapy (43, 44).

However, little is known about the effect of anti-PD-1 immunotherapy on macrophages. In an *in vivo* mouse model, Gordon et al. (45) were able to show that a PD-1/PD-L1 blockade increased macrophage-associated phagocytosis, reduced tumor growth, and increased survival. Consequently, not only the elimination of M2 macrophages appears to have a beneficial effect in immunotherapy but also the repolarization of tumorigenic M2 macrophages into tumor-suppressive M1 macrophages may serve as a target therapy in further immunotherapeutic research. In malignant melanoma, the importance of the immune system is generally accepted. Cutaneous melanomas showing regression revealed lower counts of CD4-positive T_reg_ cells and PD-1-expressing exhausted T cells. On the mRNA level, regressed melanomas showed a higher CD4 and CD8 expression (46). In addition, a more aggressive phenotype in melanomas was associated with a switch in macrophage polarization from M1 to M2 (46).

To get a more profound insight into the relation of T cells and macrophages, we calculated the amount of overall CD8^+^ stained cells in relation to the amount of CD68^+^ stained cells in BCC and compared the result to that in cSCC. Interestingly, no significant difference regarding the CD8^+^ T cell vs. CD68^+^ macrophage ratio could be found in BCC compared to cSCC. Subsequently, even though cSCC is the immunologically more active tumor, the immunological component in BCC may be primarily less relevant for the cancer development but essential for the accessibility of immune checkpoint blockade with cemiplimab or future immune-modulatory therapies in macrophage elimination and repolarization.

The adjustment of the TNM classification in colon cancer by the relevance of the individual immunogenic tumor profile may also play a distinct role in other immunological active cancer types such as NMSC. Future research is needed to develop a more profound insight into the immune cell profile of NMSC and characterize its role in checkpoint therapy and treatment response. The PD-1/PD-L1 ratio, the differentiation of CD4^+^ FOXP3^-^ T_reg_ cells, and the polarization of macrophages are only a few to name and will help accomplish the goal of a sustaining therapy to support long-term antitumor immunity.

## Data Availability Statement

The raw data supporting the conclusions of this article will be made available by the authors without undue reservation.

## Ethics Statement

The studies involving human participants were reviewed and approved by the ethics committee of the Friedrich-Alexander University Erlangen-Nuremberg. The patients/participants provided their written informed consent to participate in this study.

## Author Contributions

GF formulated the hypothesis, initiated, and conducted the study, selected the patient collective, analyzed the medical records, interpreted the immunohistochemical analyses and the whole slide imaging of the specimens, helped with cell counting, interpreted the data, and wrote the article. M-TK and CV analyzed the medical records, performed the immunohistochemical staining and the whole slide imaging of the specimens, trained the Qu-Path freeware for cell counting, interpreted the data, and contributed to the article. CG helped to interpret the immunohistochemical results and critically reviewed the article. JR helped to validate the markers and critically reviewed the article. J-EW contributed to the immunohistochemical process and data evaluation and critically reviewed the article. MK contributed to the discussion and critically reviewed the article. MW formulated the hypothesis, conducted the study, selected the patient collective, interpreted the immunohistochemical analyses and the whole slide imaging of the specimens, helped with cell counting, interpreted the data, and contributed relevantly to the article. All authors read and approved the final article.

## Funding

The project was funded by the Friedrich-Alexander University Erlangen-Nuremberg “ELAN-Anschubfinanzierung” (number: 18-12-17-1-Frohwitter).

## Conflict of Interest

The authors declare that the research was conducted in the absence of any commercial or financial relationships that could be construed as a potential conflict of interest.

## Publisher’s Note

All claims expressed in this article are solely those of the authors and do not necessarily represent those of their affiliated organizations, or those of the publisher, the editors and the reviewers. Any product that may be evaluated in this article, or claim that may be made by its manufacturer, is not guaranteed or endorsed by the publisher.
